# De novo upper tract urothelial carcinoma after renal transplantation: a single-center experience in China

**DOI:** 10.1186/s12894-023-01190-0

**Published:** 2023-02-20

**Authors:** Shixin Li, Jian Zhang, Ye Tian, Yichen Zhu, Yuwen Guo, Zhipeng Wang, Yang Yang, Guangpu Ding, Jun Lin

**Affiliations:** 1 Beijing Friendship Hospital, Capital Medical University, 95 Yongan Road, Xicheng District, Beijing, China; 2 Beijing Key Laboratory of Tolerance Induction and Organ Protection in Transplantation, 95 Yongan Road, Xicheng District, Beijing, China; 3 Qilu Hospital of Shandong University Dezhou Hospital, Dezhou, China

**Keywords:** Renal transplantation, Upper tract urothelial carcinoma, Aristolochic acid, Prognosis, Prophylactic contralateral resection

## Abstract

**Background:**

Long-term prognosis and risk factors of de novo upper tract urothelial carcinoma after renal transplantation were rarely studied. Thus, the aim of this study was to investigate the clinical features, risk factors, and long-term prognosis of de novo upper tract urothelial carcinoma after renal transplantation, especially the impact of aristolochic acid on tumor, using a large sample.

**Methods:**

106 patients were enrolled in retrospective study. The endpoints included overall survival, cancer-specific survival, bladder or contralateral upper tract recurrence-free survival. Patients were grouped according to aristolochic acid exposure. Survival analysis was performed using Kaplan–Meier curve. Log-rank test was used to compare the difference. Multivariable cox regression was conducted to evaluate the prognostic significance.

**Results:**

Median time from transplantation to development of upper tract urothelial carcinoma was 91.5 months. Cancer-specific survival rate at 1, 5, 10 years was 89.2%, 73.2%, 61.6%. Tumor staging (≥ T2), lymph node status (N +) were independent risk factors for cancer-specific death. Contralateral upper tract recurrence-free survival rate at 1, 3, 5 years was 80.4%, 68.5%, 50.9%. Aristolochic acid exposure was independent risk factor for contralateral upper tract recurrence. The patients exposed to aristolochic acid had more multifocal tumors and higher incidence of contralateral upper tract recurrence.

**Conclusion:**

Both higher tumor staging and positive lymph node status were associated with a worse cancer-specific survival in patients with post-transplant de novo upper tract urothelial carcinoma, which highlighted the importance of early diagnosis. Aristolochic acid was associated with multifocality of tumors and higher incidence of contralateral upper tract recurrence. Thus, prophylactic contralateral resection was suggested for post-transplant upper tract urothelial carcinoma, especially for patients with aristolochic acid exposure.

## Highlights

- Both higher tumor staging and positive lymph node status were associated with a worse cancer-specific survival in patients with post-transplant de novo UTUC.
- Aristolochic acid was associated with multifocality of tumors and higher incidence of contralateral upper tract recurrence.
- Prophylactic contralateral resection was suggested for post-transplant UTUC, especially for patients with aristolochic acid exposure.

## Background

Renal transplantation is the best therapeutic choice for end-stage renal disease, which exhibits a better quality of life and longer survival [1, 2]. However, long-term survival is challenged by the de novo malignancy, whose incidence is 2–4 times higher in transplant recipients than in general population [3]. Urothelial carcinoma, especially upper tract urothelial carcinoma (UTUC) is the most common malignancy after kidney transplantation in Chinese mainland and Taiwan [4–6]. However, long-term prognosis of de novo UTUC after renal transplantation was rarely studied. Although some studies revealed that aristolochic acid (AA) contained in traditional Chinese medicine might be associated with progressive renal interstitial fibrosis, chronic renal insufficiency and UTUC [7], large-sample study was still needed to further understand the prognosis and risk factors of post-transplant UTUC. Thus, this retrospective study aimed to investigate the clinical features, risk factors, and long-term prognosis of de novo UTUC after renal transplantation and focus on the impact of AA on de novo UTUC, using a large sample.

## Methods

### Inclusion and exclusion criteria

Medical records from Beijing Friendship Hospital, Capital Medical University were reviewed and totally 106 patients were enrolled. The inclusion and exclusion criteria were as follows: (a) patients who received renal transplantation between January 1,1974 and December 31, 2019, and developed de novo UTUC after transplantation, and underwent surgeries were included; (b) patients who had a urothelial carcinoma history before transplantation or developed UTUC within 6 months after transplantation were excluded; (c) de novo bladder cancer (BC) earlier than UTUC were excluded; (d) de novo UTUC occurred on allograft kidney were excluded; (e) patients with incomplete medical records were excluded.

### Data collection and grouping

Individual data was collected, including demographic data, clinical behavior, and oncological and surgical outcome. Demographic data included gender, age at renal transplantation and UTUC diagnosis, AA exposure and smoking history. The primary endpoint of this study was overall survival (OS) and cancer-specific survival (CSS). The secondary endpoint was bladder or contralateral upper tract recurrence-free survival (RFS). Tumor stage was certified according to the 8th Edition of the AJCC TNM Staging System, and the histological grade was assessed using the WHO 1973 and 2004 grading system. Patients were grouped into AA group and non-AA group according to the history of exposure to Chinese herbs containing AA before renal transplantation.

### Statistical analysis

IBM SPSS version 22 software (IBM Corporation, Armonk, NY, USA) was applied for statistical analysis. Measurement data was expressed as mean [standard deviation (SD)] for normal distribution or median (p25-p75) for skewed distribution. Mann Whitney u test was used for continuous variables, and *χ*2-test or Fisher’s exact test was used for categorical variables. Kaplan–Meier survival analysis was performed to estimate OS, CSS and RFS. The log-rank test was used to compare the difference between two groups. Multivariable cox regression was conducted to evaluate the prognostic significance of each variable. *p* < 0.05 was considered statistically significant.

## Results

### Patients’ information and clinical features

**Fig. 1:**
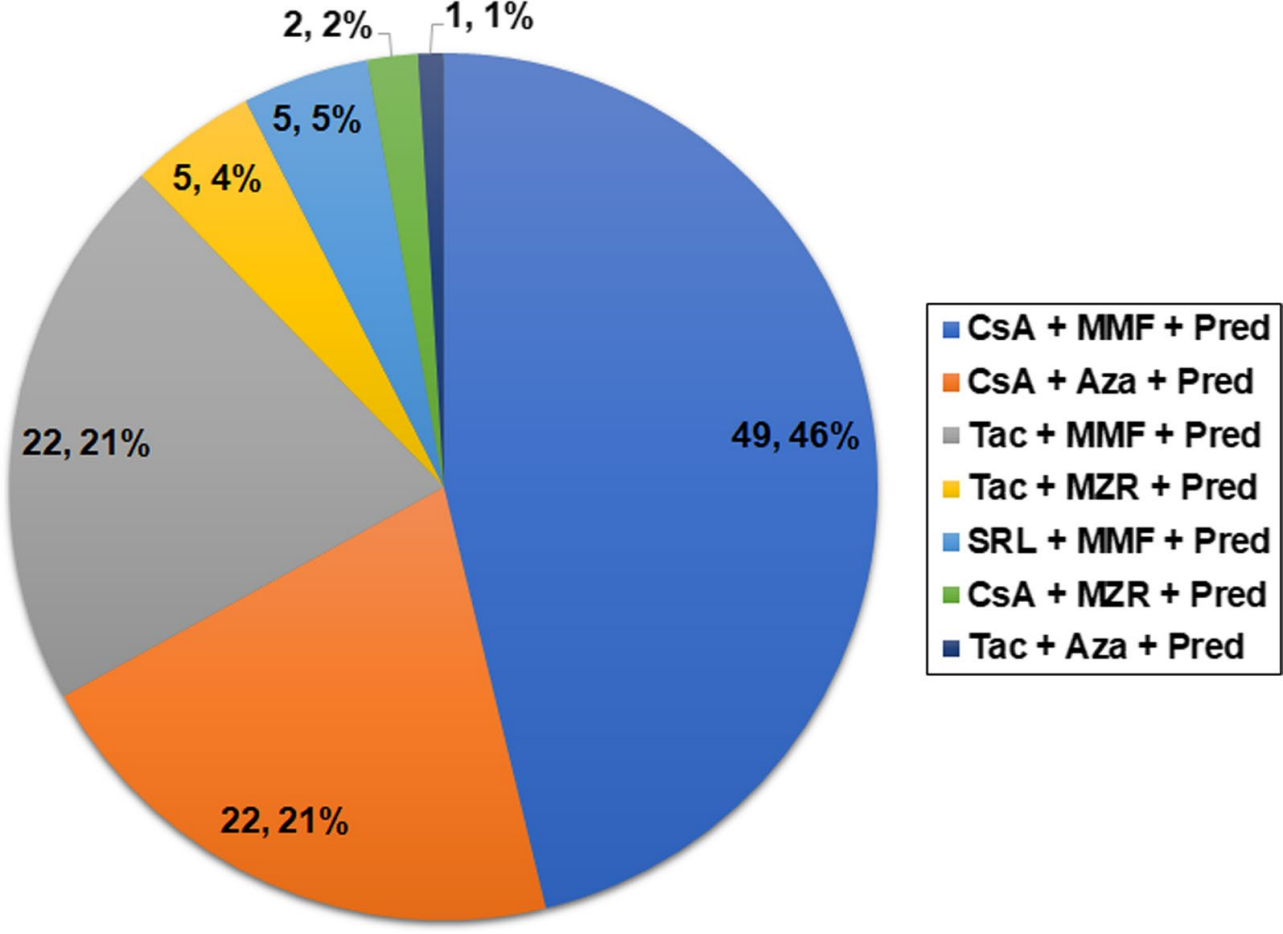
Immunosuppressive regimen of patients with de novo UTUC after renal transplantation. *CsA* Cyclosporin A, *Tac* Tacrolimus, *SRL* Sirolimus; *MMF* Mycophenolate mofetil, *Aza* Azathioprine, *MZR* Mizoribine, *Pred* Prednisone

The median follow-up period from development of UTUC was 96 (55–148) months. The median age at time point of renal transplantation and development of UTUC was 48.5 (44–54.25) and 57 (51–62) years old, respectively. The time from transplantation to development of UTUC was shown in Table 1, with a median time of 91.5 (48–143.75) months. Male to female ratio was approximately 1/5 (17/89). Only three patients received a second transplant. Totally, 81 (76.4%) patients had a history of AA exposure that they intermittently took AA-containing herbs for a considerable time according to the package insert. Only 4 (3.8%) patients had smoking history. None of the patients had a history of alcohol abuse. Familial clustering was not observed in this study. Cyclosporin A (CsA) combined with mycophenolate mofetil (MMF) and prednisone (Pred) (*n* = 49, 46%), CsA combined with azathioprine (Aza) and Pred (*n* = 22, 21%), tacrolimus (Tac) combined with MMF and Pred (*n* = 22, 21%) were predominant immunosuppressive regimens (Fig. 1).

**Table 1:** Clinical characteristics of patients with de novo UTUC

Variable	Number of patients, n (%)
*Interval time from transplantation to development of UTUC*	
< 1 year	1 (0.9%)
1–5 years	35 (33%)
5–10 years	31 (29.2%)
10–15 years	25 (23.6%)
> 15 years	14 (13.2%)
*Tumor staging*	
Ta	6 (5.7%)
T1	46 (43.4%)
T2	17 (16%)
T3	28 (26.4%)
T4	9 (8.5%)
*Lymph node staging*	
N +	6 (5.7%)
N0	100 (94.3%)
*Tumor grade*	
G1/G2	56 (52.8%)
G3	50 (47.2%)
Multifocality	67 (63.2%)
*Tumor location*	
Bilateral tumor	7 (6.6%)
Bilateral upper tract	1 (0.9%)
Bilateral upper tract and bladder	6 (5.7%)
Unilateral tumor	99 (93.4%)
Renal pelvis only	18 (18.2%)
Ureter only	27 (27.3%)
Renal pelvis and ureter	27 (27.3%)
Renal pelvis and bladder	5 (5.1%)
Ureter and bladder	5 (5.1%)
Renal pelvis, ureter and bladder	17 (17.2%)

*UTUC* Upper tract urothelial carcinoma

Hematuria was the most frequent initial symptom of UTUC, accounting for 82.1%, including gross hematuria in 72 patients and microscopic hematuria in 15 patients. Hydronephrosis was the most common manifestation, which was observed in 90 patients, with an incidence of 80.2%, including 13 patients with asymptomatic hydronephrosis. Low back pain was found in 23 patients, accounting for 21.7%. 2 patients found tumor without hematuria, low back pain or hydronephrosis in a routine check-up.

**Fig. 2:**
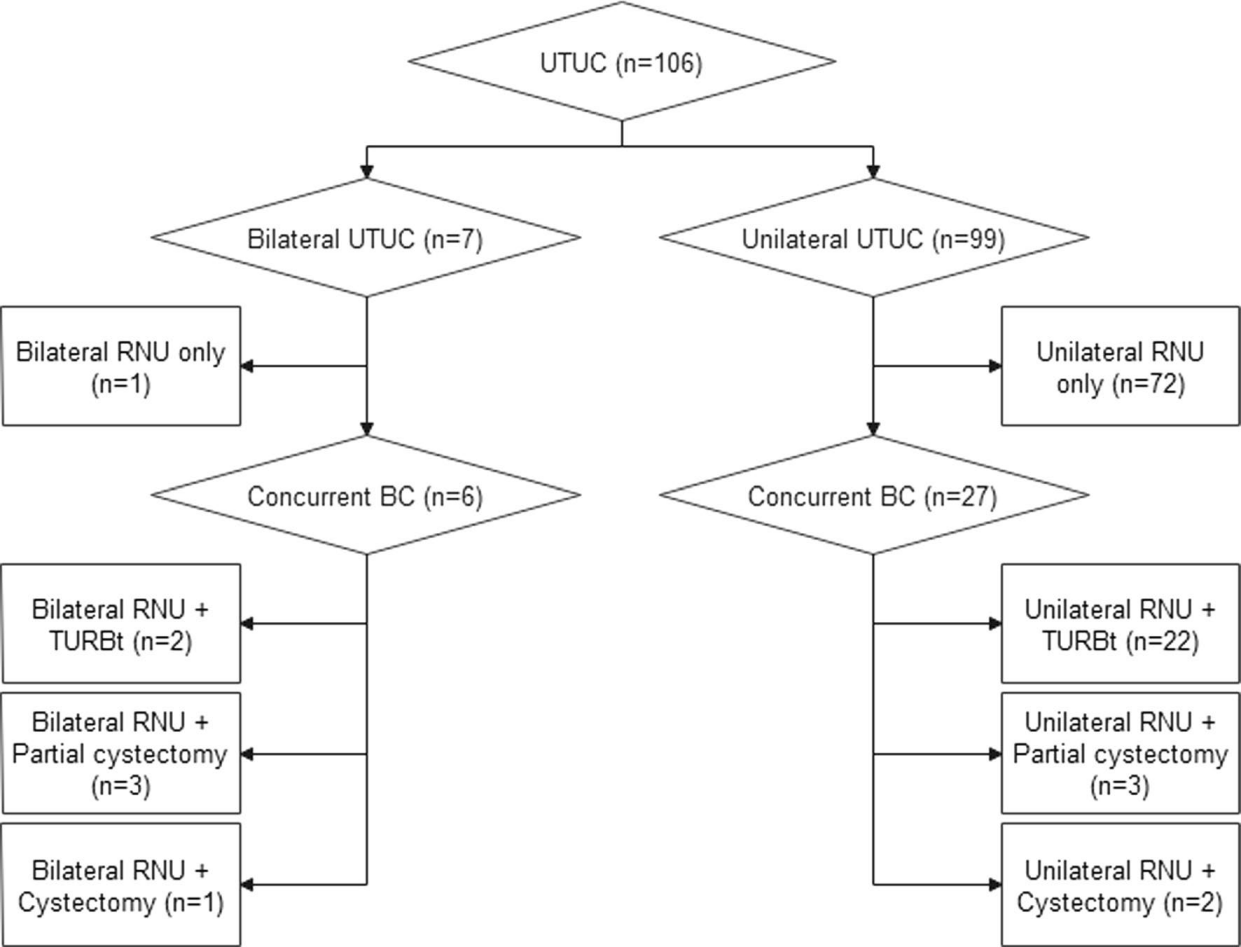
Surgical protocol for UTUC. *UTUC* Upper tract urothelial carcinoma, *RNU* Radical nephroureterectomy, *BC* Bladder cancer, *TURBt* Transurethral resection of bladder tumor

Surgical protocol was included in Fig. 2. 7 patients with synchronous bilateral UTUC underwent simultaneous bilateral RNU and 99 patients with unilateral UTUC underwent unilateral RNU. 1 patient removed unilateral kidney due to renal tuberculosis before renal transplantation. 4 patients removed unilateral kidney before development of UTUC due to hydronephrosis with low back pain.

The clinical characteristics of patients with de novo UTUC were shown in Table 1. Non-muscle-invasive tumors (< T2) accounted for 49.1% (*n* = 52), while muscle-invasive tumors (≥ T2) accounted for 50.9% (*n* = 54). The histological tumor grade of G1, G2 and G3 accounted for 2.8% (*n* = 3), 50.0% (*n* = 53), and 47.2% (*n* = 50), respectively. Multifocal tumors were observed in 67 (63.2%) patients.

### Oncological outcome

**Fig. 3:**
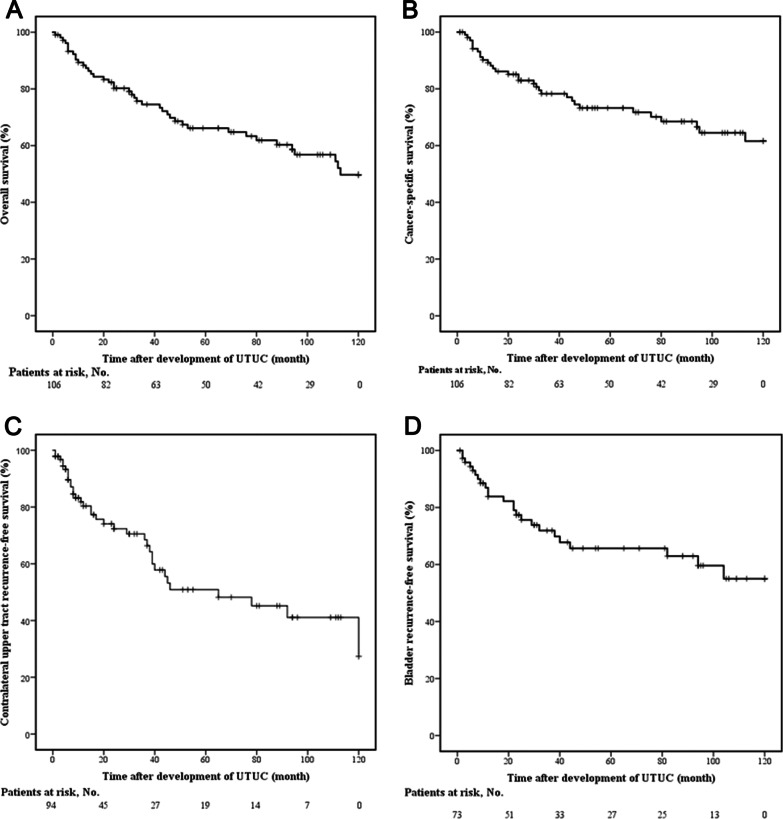
Survival of patients with de novo UTUC after renal transplantation. **A** Estimated Kaplan–Meier curve representing OS; **B** Estimated Kaplan–Meier curve representing CSS; **C** Estimated Kaplan–Meier curve representing contralateral upper tract RFS; **D** Estimated Kaplan–Meier curve representing bladder RFS. *OS* Overall survival, *CSS* Cancer-specific survival, *RFS* Recurrence-free survival

Totally 41 patients died during the follow-up period, with an overall mortality rate of 38.7%. The time from development of UTUC to patients’ death ranged from 25 days to 193 months. The OS and CSS rate at 1, 5, 10 years were 88.3%, 66.1%, 49.7% (Fig. 3A) and 89.2%, 73.2%, 61.6% (Fig. 3B), respectively. Univariable cox regression analysis showed that tumor staging (≥ T2) (HR = 4.488, *p* < 0.001), lymph node status (N +) (HR = 23.486, *p* < 0.001) and tumor grade (G3) (HR = 2.635, *p* = 0. 01) were significant risk factors for cancer-specific death. Multivariate cox regression analysis showed that tumor staging (≥ T2) (HR = 3.234, *p* = 0.009), lymph node status (N +) (HR = 12.91, *p* < 0.001) were independent risk factors for cancer-specific death (Table 2).

**Table 2:** Risk factors for cancer-specific death of patients with de novo UTUC

Variable	Univariate analysis		Multivariate analysis	
	HR (95% CI)	*p*-value	HR (95% CI)	*p*-value
*Gender*				
Male	1 (Ref.)			
Female	0.937 (0.327–2.690)	0.904		
*Age*				
≤ 65y	1 (Ref.)			
> 65y	1.255 (0.481–3.276)	0.643		
*AA exposure*				
No	1 (Ref.)			
Yes	1.231 (0.471–3.214)	0.671		
*Tumor staging*				
< T2	1 (Ref.)		1 (Ref.)	
≥ T2	4.488 (1.996–10.093)	< 0.001	3.234 (1.342–7.795)	0.009
*Lymph node staging*				
N0	1 (Ref.)		1 (Ref.)	
N +	23.486 (7.815–70.585)	< 0.001	12.91 (4.217–39.51)	< 0.001
*Tumor grade*				
G1/G2	1 (Ref.)		1 (Ref.)	
G3	2.635 (1.264–5.493)	0.01	1.416 (0.607–3.301)	0.421
*Multifocality*				
No	1 (Ref.)			
Yes	1.518 (0.678–3.397)	0.31		
*Concomitant BC*				
No	1 (Ref.)			
Yes	1.878 (0.918–3.842)	0.084		

*AA* Aristolochic acid, *BC* Bladder cancer, *CI* Confidence interval, *HR* Hazard ratio, *Ref*. Reference

Totally 94 patients were included for analysis of contralateral recurrence, except 7 patients with synchronous bilateral UTUC, 1 patient with contralateral resection due to renal tuberculosis, and 4 patients with contralateral resection before development of UTUC. Contralateral recurrence was found in 37.2% (35/94) of patients, with a median recurrent time of 15 (6–39) months. The contralateral upper tract RFS rate at 1, 3, 5 years was 80.4%, 68.5%, and 50.9%, respectively (Fig. 3C). Univariable cox regression analysis showed that AA exposure (HR = 4.817, *p* = 0.031), multifocality (HR = 2.212, *p* = 0.041) and tumor location of pelvis and ureter (HR = 3.114, *p* = 0.040) were significant risk factors for contralateral upper tract recurrence. Multivariate cox regression analysis showed that AA exposure (HR = 4.714, *p* = 0.037) was independent risk factor for contralateral upper tract recurrence (Table 3).

**Table 3:** Risk factors for contralateral recurrence of patients with de novo UTUC

Variable	Univariate analysis		Multivariate analysis	
	HR (95% CI)	*p*-value	HR (95% CI)	*p*-value
*Gender*				
Male	1 (Ref.)			
Female	0.519 (0.158–1.703)	0.279		
*Age*				
≤ 65y	1 (Ref.)			
> 65y	0.910 (0.462–1.792)	0.785		
*AA exposure*				
No	1 (Ref.)		1 (Ref.)	
Yes	4.817 (1.154–20.16)	0.031	4.714 (1.096–20.272)	0.037
*Tumor staging*				
< T2	1 (Ref.)			
≥ T2	1.724 (0.801–3.710)	0.163		
*Lymph node staging*				
N0	1 (Ref.)			
N +	0.047 (0.000–8582.93)	0.621		
*Tumor grade*				
G1/G2	1 (Ref.)			
G3	0.796 (0.376–1.687)	0.552		
*Multifocality*				
No	1 (Ref.)		1 (Ref.)	
Yes	2.212 (1.033–4.737)	0.041	1.196 (0.438–3.271)	0.727
*Concomitant BC*				
No	1 (Ref.)			
Yes	1.786 (0.874–3.651)	0.112		
*Tumor location*				
Renal pelvis only	1 (Ref.)		1 (Ref.)	
Ureter only	1.676 (0.533–5.273)	0.377	2.132 (0.665–6.836)	0.203
Pelvis and ureter	3.114 (1.052–9.215)	0.040	2.861 (0.787–10.396)	0.110

*AA* Aristolochic acid, *BC* Bladder cancer, *CI* Confidence interval, *HR* Hazard ratio, *Ref.* Reference

Totally 73 patients were included for analysis of bladder recurrence, except 33 patients with initially concurrent BC. Bladder recurrence was found in 32.9% (24/73) of patients, with a median recurrent time of 20 (7.25–36.50) months. The bladder RFS rate at 1, 3, 5 years was 83.8%, 71.9%, and 65.7%, respectively (Fig. 3D). There was no significant risk factor for bladder recurrence according to univariable cox regression analysis (Table 4).

**Table 4:** Risk factors for bladder recurrence of patients with de novo UTUC

Variable	Univariate analysis	
	HR (95% CI)	*p*-value
*Gender*		
Male	1 (Ref.)	
Female	1.246 (0.423–3.671)	0.690
*Age*		
≤ 65y	1 (Ref.)	
> 65y	0.787 (0.269–2.309)	0.663
*AA exposure*		
No	1 (Ref.)	
Yes	1.296 (0.483–3.477)	0.606
*Tumor staging*		
< T2	1 (Ref.)	
≥ T2	1.299 (0.574–2.939)	0.531
*Lymph node staging*		
N0	1 (Ref.)	
N +	0.047 (0.001–50,788.947)	0.665
*Tumor grade*		
G1/G2	1 (Ref.)	
G3	1.092 (0.474–2.519)	0.836
*Multifocality*		
No	1 (Ref.)	
Yes	1.138 (0.509–2.543)	0.753
*Tumor location*		
Renal pelvis only	1 (Ref.)	
Ureter only	1.447 (0.523–4.004)	0.477
Pelvis and ureter	0.965 (0.372–2.506)	0.942

*AA* Aristolochic acid, *CI* Confidence interval, *HR* Hazard ratio, *Ref.* Reference

### Impact of AA on de novo UTUC

**Fig. 4:**
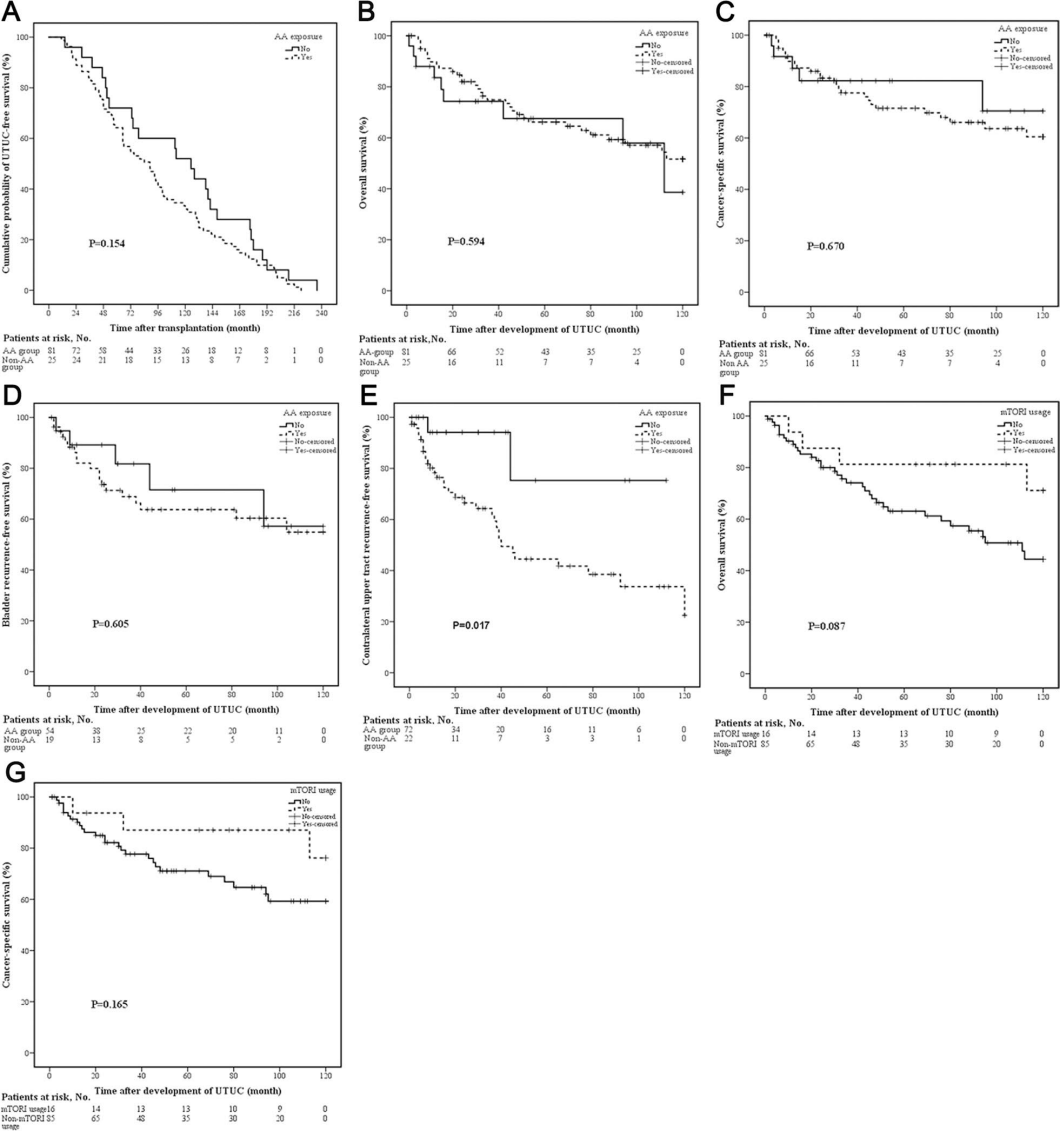
Survival of patients with de novo UTUC after renal transplantation stratified by AA exposure or conversion to mTOR inhibitor. **A** No statistical difference was observed in UTUC-free survival stratified by AA exposure; **B** No statistical difference was observed in OS stratified by AA exposure; **C** No statistical difference was observed in CSS stratified by AA exposure; **D** No statistical difference was observed in bladder RFS stratified by AA exposure; **E** The contralateral upper tract RFS rate in AA group was lower than that in non-AA group (*p* = 0.017); **F** No statistical difference was observed in OS stratified by conversion to mTOR inhibitor; **G** No statistical difference was observed in CSS stratified by conversion to mTOR inhibitor. *UTUC* Upper tract urothelial carcinoma; *OS* Overall survival, *CSS* Cancer-specific survival, *RFS* Recurrence-free survival, *AA* Aristolochic acid, *mTOR* Mammalian target of rapamycin

Clinical characteristics of patients in AA group and non-AA group were shown in Table 5. The patients exposed to AA had more multifocal tumors (69.1% vs. 44.0%, *p* = 0.023) and higher contralateral upper tract recurrent rate (45.8% vs. 9.1%, *p* = 0.002). We did not observe any statistical difference between the two groups with regard to UTUC-free survival, OS, CSS, and bladder RFS (Fig. 4A–D). The contralateral upper tract RFS rate in AA group was lower than that in non-AA group (Fig. 4E).

**Table 5:** Clinical characteristics of patients in AA group and non-AA group

Variable	AA group	non-AA group	*p*-value
Number of patients, *n* (%)	81 (76.4%)	25 (23.6%)	
Age at transplantation, years	48 (45–52)	51 (36–55)	0.985
Age at UTUC, years	56 (51–60)	59 (44–68)	0.350
Time to UTUC, months	89 (47.5–134)	125 (53–177.5)	0.132
Male/female ratio	10/71	7/18	0.120
Smoking history, *n* (%)	3 (3.7%)	1 (4.0%)	0.946
Symptoms, *n* (%)			
Ipsilateral hydronephrosis	69 (85.2%)	21 (84.0%)	1.00
Hematuria	67 (82.7%)	20 (80%)	0.991
Tumor staging, *n* (%)			0.135
T ≥ 2	38 (46.9%)	16 (64.0%)	
T < 2	43 (53.1%)	9 (36.0%)	
Lymph node staging, *n* (%)			0.154
N +	2 (2.5%)	3 (12.0%)	
N0	79 (97.5%)	22 (88.0%)	
Tumor grade, *n* (%)			0.142
G3	35 (43.2%)	15 (60.0%)	
G1/G2	46 (56.8%)	10 (40.0%)	
Multifocality, *n* (%)	56 (69.1%)	11 (44.0%)	0.023
Location, *n* (%)			0.104
Renal pelvis only	19 (23.5%)	5 (20.0%)	
Ureter only	21 (25.9%)	12 (48.0%)	
Pelvis and ureter	41 (50.6%)	8 (32.0%)	
Concomitant BC, *n* (%)	27 (33.3%)	6 (24.0%)	0.378
Contralateral recurrence, *n* (%)^*^	33 (45.8%)	2 (9.1%)	0.002
Bladder recurrence, *n* (%)^#^	19 (35.2%)	5 (26.3%)	0.479

^*^Totally 94 patients were included for analysis of contralateral recurrence, except 7 patients with synchronous bilateral UTUC, 1 patient with contralateral resection due to renal tuberculosis, and 4 patients with contralateral resection before development of UTUC; #: Totally 73 patients were included for analysis of bladder recurrence, except 33 patients with initially concurrent BC

*AA* Aristolochic acid, *UTUC* Upper tract urothelial carcinoma, *BC* Bladder cancer

### Efficacy of conversion to rapamycin

Totally 16 of 101 patients received a conversion from CNIs to mTOR inhibitor after development of UTUC. Figure 4F–G revealed no significant difference of either OS or CSS between patients with and without conversion to mTOR inhibitor.

## Discussion

Compared with general population, renal transplant recipients were at higher risk of developing urothelial carcinomas [8, 9]. There were obviously geographic and gender differences in the prevalence of UTUC after renal transplantation. Several studies from Chinese mainland and Taiwan revealed an incidence of 0.97% to 6.47% [6, 9–13], while the incidence in Western countries varied from 0.04 to 0.3% [14–16]. Besides, post-transplant UTUC was more prevalent in female patients [6, 13, 17, 18]. In this study, 89 patients with UTUC were female, with a male to female ratio of approximately 1/5, which was in accordance with previous studies.

AA had been well investigated as a cause of UTUC, of which the mechanism was that AA metabolites induced mutations in p53 tumor suppressor gene, and eventually led to the activation of proto-oncogenes and tumor induction [19]. AA DNA adduct was still detected in renal tissue even if over 20 years after cessation [20]. The carcinogenic effect of AA could persist for many years and was associated with usage dose and time, which rendered patients prone to develop UTUC [21]. Differences in both incidence and gender of patients with post-transplant UTUC between Asian countries and Western countries seemed to be associated with AA exposure. In this study, 76.4% patients had a history of exposure to AA, which might be the major reason for developing UTUC. Although no statistical difference of UTUC-free survival was observed between two groups, we did observe an earlier development of UTUC in AA group by the survival curve that the limited sample in non-AA group might result in the bias. Furthermore, this study demonstrated that AA contributed to both multifocality of tumors and contralateral upper tract recurrence, which was consistent with previous study [22] and provided evidence for prophylactic contralateral resection. It was noteworthy that the smoking history, which was a proven risk factor for both BC and UTUC [23], was not as important as AA exposure for post-transplant UTUC. Besides, previous study demonstrated that alcohol consumption might be an independent risk factor for UTUC and the risk threshold was > 15 g of alcohol consumption per day [24]. However, alcohol consumption might not be a risk factor in this study, because none of the patients had a history of alcohol abuse.

In this study, both long-term OS and CSS seemed to be similar with other studies containing the general population [25, 26], which was inconsistent with the previous perception that these should be lower in renal transplant recipients. We considered that might owe to an earlier, more active, and comprehensive treatment in our center. In addition, we found that both higher tumor staging and positive lymph node status were associated with a worse CSS in patients with post-transplant de novo UTUC, which was consistent with the recent study [27] and highlighted the importance of early diagnosis.

Hematuria and hydronephrosis were common symptoms in general patients with UTUC, which was also observed in renal transplant cohort. In this study, hematuria or native hydronephrosis was observed in most of patients, which could be a warning of UTUC after renal transplantation, especially for patients with de novo manifestation. Several reasons were attributed to native hydronephrosis, while UTUC was the most common and noteworthy. Previous study showed a strong correlation between native hydronephrosis and UTUC [9]. In this study, many patients showed native hydronephrosis pre-operatively, which was in accordance with the previous study. In addition, we found that native hydronephrosis might be the only manifestation in a considerable proportion of the patients, which should not be ignored.

Immunosuppressive agents played a vital role in the development of cancers after renal transplantation, which was proved to have direct oncogenic effects [3]. Calcineurin inhibitors (CNIs) such as cyclosporine and tacrolimus could induce transforming growth factor β (TGF-β) hyperexpression in mice, which might promote tumor growth and metastatic progression [28]. Immunosuppressive agents also impaired immune surveillance and promoted the occurrence of virus-associated malignancies [29]. Recently, mammalian target of rapamycin (mTOR) inhibitor revealed an anti-tumor effect in post-transplant recipients with switching regimen, however, there was also reversed opinion about this protective effect [30, 31]. In this study, Kaplan–Meier survival curve revealed that both OS and CSS seemed to be better in patients with conversion to mTOR inhibitor, however, there was no statistical significance, which might be attributed to the limited number of patients with conversion regimen.

Previous studies demonstrated that renal transplant recipients with UTUC were prone to contralateral or bladder recurrence, and remained at risk of recurrence for many years after surgery [32]. In this study, we observed a high incidence of contralateral upper tract recurrence, which indicated the necessity of prophylactic contralateral resection for post-transplant UTUC, especially for patients with AA exposure, which was found to be an independent risk factor for contralateral recurrence. A previous study from our center had demonstrated the benefit from prophylactic resection, using a small sample [33]. More recently, Zhang et al. found that simultaneous bilateral radical nephroureterectomy contributed to improve survival compared with unilateral radical nephroureterectomy [13]. In our experience, prophylactic contralateral resection could be safely performed approximately 3 months after the previous surgery.

There were limitations in this study. Firstly, data selection bias was existed due to the retrospective nature. Secondly, AA exposure was determined by the medication history without a definite description of dosage or duration, lacking of reliable markers.

## Conclusions

Both higher tumor staging and positive lymph node status were associated with a worse CSS in patients with post-transplant de novo UTUC, which highlighted the importance of early diagnosis. AA was associated with multifocality of tumors and higher incidence of contralateral upper tract recurrence. Prophylactic contralateral resection was suggested for post-transplant UTUC, especially for patients with AA exposure.

## Data Availability

The datasets generated and/or analysed during the current study are not publicly available due to privacy restrictions but are available from the corresponding author on reasonable request.

## Abbreviations

AA: Aristolochic acid
Aza: Azathioprine
BC: Bladder cancer
CNIs: Calcineurin inhibitors
CSS: Cancer-specific survival
CsA: Cyclosporin A
MMF: Mycophenolate mofetil
mTOR: Mammalian target of rapamycin
MZR: Mizoribine
OS: Overall survival
Pred: Prednisone
RFS: Recurrence-free survival
RNU: Radical nephroureterectomy
SD: Standard deviation
SRL: Sirolimus
Tac: Tacrolimus
TGF-β: Transforming growth factor β
TURBt: Transurethral resection of bladder tumor
UTUC: Upper tract urothelial carcinoma
